# Air Pollution and Preterm Birth in the U.S. State of Georgia (2002–2006): Associations with Concentrations of 11 Ambient Air Pollutants Estimated by Combining Community Multiscale Air Quality Model (CMAQ) Simulations with Stationary Monitor Measurements

**DOI:** 10.1289/ehp.1409651

**Published:** 2015-10-20

**Authors:** Hua Hao, Howard H. Chang, Heather A. Holmes, James A. Mulholland, Mitch Klein, Lyndsey A. Darrow, Matthew J. Strickland

**Affiliations:** 1 Department of Environmental Health, and; 2 Department of Biostatistics and Bioinformatics, Rollins School of Public Health, Emory University, Atlanta, Georgia, USA; 3 Department of Physics, University of Nevada-Reno, Reno, Nevada, USA; 4 School of Civil and Environmental Engineering, Georgia Institute of Technology, Atlanta, Georgia, USA; 5 Department of Epidemiology, Rollins School of Public Health, Emory University, Atlanta, Georgia, USA

## Abstract

**Background:**

Previous epidemiologic studies suggest associations between preterm birth and ambient air pollution.

**Objective:**

We investigated associations between 11 ambient air pollutants, estimated by combining Community Multiscale Air Quality model (CMAQ) simulations with measurements from stationary monitors, and risk of preterm birth (< 37 weeks of gestation) in the U.S. state of Georgia.

**Methods:**

Birth records for singleton births ≥ 27 weeks of gestation with complete covariate information and estimated dates of conception between 1 January 2002 and 28 February 2006 were obtained from the Office of Health Indicators for Planning, Georgia Department of Public Health (n = 511,658 births). Daily pollutant concentrations at 12-km resolution were estimated for 11 ambient air pollutants. We used logistic regression with county-level fixed effects to estimate associations between preterm birth and average pollutant concentrations during the first and second trimester. Discrete-time survival models were used to estimate third-trimester and total pregnancy associations. Effect modification was investigated by maternal education, race, census tract poverty level, and county-level urbanicity.

**Results:**

Trimester-specific and total pregnancy associations (p < 0.05) were observed for several pollutants. All the traffic-related pollutants (carbon monoxide, nitrogen dioxide, PM2.5 elemental carbon) were associated with preterm birth [e.g., odds ratios for interquartile range increases in carbon monoxide during the first, second, and third trimesters and total pregnancy were 1.005 (95% CI: 1.001, 1.009), 1.007 (95% CI: 1.002, 1.011), 1.010 (95% CI: 1.006, 1.014), and 1.011 (95% CI: 1.006, 1.017)]. Associations tended to be higher for mothers with low educational attainment and African American mothers.

**Conclusion:**

Several ambient air pollutants were associated with preterm birth; associations were observed in all exposure windows.

**Citation:**

Hao H, Chang HH, Holmes HA, Mulholland JA, Klein M, Darrow LA, Strickland MJ. 2016. Air pollution and preterm birth in the U.S. state of Georgia (2002–2006): associations with concentrations of 11 ambient air pollutants estimated by combining Community Multiscale Air Quality Model (CMAQ) simulations with stationary monitor measurements. Environ Health Perspect 124:875–880; http://dx.doi.org/10.1289/ehp.1409651

## Introduction

Preterm birth is a well-known predictor of infant morbidity and mortality as well as increased long-term morbidity in adulthood (Goldenberg et al. 2008; Hille et al. 2008; van Lunenburg et al. 2013; Swamy 2012). Several epidemiologic studies suggest associations between ambient air pollutant concentrations and preterm birth, although there are inconsistencies regarding the gestational windows of greatest susceptibility and the specific pollutants most strongly associated with preterm birth. Some of this heterogeneity is likely attributable to differences in exposure assignment methods and epidemiologic modeling approaches across studies (Shah et al. 2011; Stieb et al. 2012).

Many previous studies have used measurements from central-site monitors to assign exposures (Shah et al. 2011), which may have limited spatial representativeness, particularly for primary pollutants such as carbon monoxide (CO) and nitrogen dioxide (NO_2_), which are known to be spatially heterogeneous (Thurston et al. 2009). Furthermore, monitors are often located in urban areas, and measurements from these monitors may poorly reflect ambient air pollutant concentrations in rural areas. Alternative methods for estimating ambient air pollutant concentrations include land-use regression, satellite-based prediction, and chemical transport models such as the Community Multiscale Air Quality model (CMAQ) (Özkaynak et al. 2013). In recent work we combined the daily 12-km ambient air quality CMAQ outputs with observations from a network of ambient air quality monitors throughout the U.S. state of Georgia (Friberg et al. 2016). This fusion approach retains the high spatial and temporal coverage afforded by the CMAQ model while seeking to reduce biases that may be present in the CMAQ simulations (Lewis et al. 2011). Recent epidemiological work employing CMAQ estimates include investigation of associations with adverse health outcomes including asthma (Sacks et al. 2014), allergy (Weir et al. 2013), myocardial infarction (Rich et al. 2013), hypertension (Männistö et al. 2015), low birth weight (Vinikoor-Imler et al. 2014), and mortality (Boldo et al. 2011). In the present study, we investigated associations between preterm birth and air pollutants by combing (fusing) the CMAQ estimates with data from stationary monitors for 11 ambient air pollutants to improve exposure estimation. We present trimester-specific and total pregnancy associations for each pollutant and investigate effect modification of these associations by maternal education, maternal race, urbanicity, and census tract poverty level.

## Methods

### Data and Outcome Assessment

Birth certificate data were obtained from the Office of Health Indicators for Planning, Georgia Department of Public Health. We included singleton pregnancies with gestational lengths of 27–42 weeks and an estimated date of conception during 1 January 2002 through 28 February 2006 (*n* = 588,886). Gestational age was defined according to date of last menstrual period, and date of conception was estimated by adding 14 days to the date of last menstrual period. By limiting to births ≥ 27 weeks we ensured that all births were followed for the entirety of the first and second trimesters. Preterm birth was defined as a birth < 37 weeks of gestation. We further excluded births where *a*) maternal residential address at delivery was unsuccessfully geocoded to the 2000 census block group (*n* = 63,162), *b*) birth weight < 400 grams (*n* = 2,784), *c*) mother’s age < 15 years or > 44 years (*n* = 5,663), *d*) one or more identified congenital anomaly (*n* = 2,210), and *e*) preterm births that had a procedure code for induction of labor (*n* = 3,409). The final data set contained 511,658 pregnancies. Urbanicity for each pregnancy was assigned using a dichotomous variable based on the U.S. National Center for Health Statistics classification scheme according to county of maternal residence (Ingram and Franco 2012). Counties in metropolitan statistical areas with > 1 million people were considered “large metropolitan counties”; all other counties were grouped together as “medium metropolitan,” “small metropolitan,” or “nonmetropolitan” counties. This study was approved by the Institutional Review Board of Emory University (IRB No. 45413) and a waiver of informed consent was granted.

### Ambient Air Pollutant Concentrations

Ambient air pollutant concentrations were estimated by fusing 12-km by 12-km outputs from the U.S. EPA’s CMAQ model with ambient air pollutant concentration measurements from Air Quality System stationary monitors. The number of monitors ranged from 5 for CO to 42 for PM_2.5_; the distribution of monitor locations (see Figure S1) and the frequency of measurements also varied across pollutants. Observations provide reliable temporal trends at and near monitors, and CMAQ simulations provide spatially rich information that is less reliable temporally. Our approach to combining these data involves three steps (Friberg et al. 2016). First, annual average CMAQ fields are used to interpolate daily observations, with the temporal variation of the derived fused fields driven by the temporal variation in observations. Second, daily CMAQ fields are scaled to annual average observations, with the temporal variation of the derived fused fields driven by the temporal variation in the CMAQ fields. Third, these two fused fields are combined to maximize prediction of temporal variance over space. For each grid cell, we calculated daily concentrations for 1-hr maximum CO, NO_2_, and sulfur dioxide (SO_2_), 8-hr maximum ozone (O_3_), 24-hr average particulate matter ≤ 10 and ≤ 2.5 μm in diameter (PM_10_, PM_2.5_), and the PM_2.5_ components sulfate (SO_4_^2–^), nitrate (NO_3_^–^), ammonium (NH_4_^+^), elemental carbon (EC), and organic carbon (OC). Pollutant concentrations for each pregnancy were assigned by linking the geocoded maternal census block centroid at delivery to the associated 12-km by 12-km grid cell based on the block group centroid. (For further details about the CMAQ data fusion method, see Figure S2.)

To evaluate model performance we created scatterplots to compare the daily pollutant measurements at the monitors with the estimated concentrations at the corresponding grids, with the relationships summarized using Pearson correlation coefficients. Leave-one-monitor-out cross-validation was used to estimate the Pearson correlation coefficient between the estimates and the measurements from the monitor that was held out of the model fitting. Fractional error—the ratio of the error of the estimate to the measured concentration—was calculated for each monitor in the leave-one-monitor-out cross-validation, and these fractional errors were averaged to calculate the mean fractional error.

### Statistical Analysis

We used logistic regression to estimate associations between preterm birth (< 37 weeks of gestation) and average pollutant concentrations during the first trimester (date of conception through gestational week 13) and second trimester (weeks 14–26 of gestation) (model 1), and a discrete-time survival model to estimate associations during the third trimester and total pregnancy (model 2) to preclude bias due to differing exposure averaging periods between preterm and full-term births (Chang et al. 2013). Specifically,


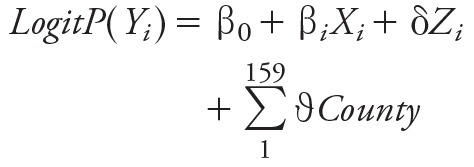
[1]


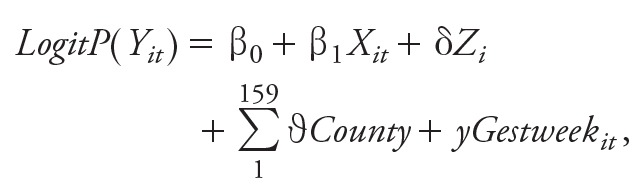
, [2]

where *Y_i_* in model 1 is the dichotomous outcome indicating whether pregnancy *i* ended in a preterm birth (gestational age < 37 weeks), and *Y_it_* in model 2 indicates whether a birth occurred during gestational week *t* for pregnancy *i*. The β*_i_* parameter is the estimate of the association with average pollutant concentrations in both model 1 and 2. In model 1, *X_i_* is the average pollution exposure during the first or second trimester for pregnancy *i*, and in model 2 *X_it_* is the time-varying average pollution exposure for pregnancy *i* between week 27 and the end of week *t* for the third-trimester model or between week 1 and the end of week *t* for the total pregnancy model. δ is a vector of parameters for the individual-level covariates (*Z_i_*) for pregnancy *i*. ϑ is a vector of fixed effects for the 159 counties, and γ in model 2 allows the baseline hazard to vary by gestational week.

We controlled for maternal education (less than 9th grade, 9th–12th grade, high school graduate, some college or higher), race (white; African American; Asian; Native American or Alaskan; Native Hawaiian or Pacific Islands; multiracial), smoking (dichotomous variable for self-reported tobacco use during pregnancy), and long-term trend using a natural cubic spline on conception date with 5 degrees of freedom (df; 1 per year) to account for the gradual increasing rate of preterm birth and decreasing concentrations of pollutants during the study period (Peng et al. 2006). Data on Hispanic ethnicity were not available. Sensitivity analyses examined additional control using 9 (2 per year) or 17 (4 per year) df on conception date. Other maternal variables (number of previous births, maternal self-reported alcohol use, maternal age, and Medicaid payment for delivery) were included in the initial regression models, but because these factors changed the estimated coefficients for the associations between air pollutant concentrations and preterm birth by < 10% they were not retained in the final models. To investigate effect modification of the association between air pollution and preterm birth we stratified the data by maternal education (equal or less than high school vs. some college or higher), race (African American vs. all other), county-level urbanicity (large metropolitan counties vs. medium, small, and nonmetropolitan counties), and tertiles of year 2000 census tract percentage below poverty, with separate regression models fit for each stratum.

Odds ratios (ORs) are presented per interquartile-range (IQR) increase in pollutant concentration. Because the estimated association between air pollution and preterm birth is largely driven by within-county contrasts (due to the county-level fixed effects in the regression model), and given that each county has its own IQR for a given pollutant, we set the overall IQRs used for presenting the health associations as being equal to the median of the 159 county-specific IQRs. Average pollutant levels during pregnancy were used to calculate the IQRs, and we used the same IQR values to present results from the first-, second-, and third-trimester and total pregnancy models.

## Results

The study cohort consisted of 511,658 singleton births across 159 Georgia counties, 47,321 (9.3%) of whom were preterm. Compared with mothers of full-term births, mothers of preterm births were more likely to reside in a large metropolitan county, be of African-American race, have a low education level, use tobacco products, and live in more impoverished census tracts (all *p*-values < 0.0001 for the differences among all categories of each characteristic) (Table 1). Statewide, the proportion of births born preterm increased slightly over the study period (from 8.9% to 9.6% for conceptions during 1 January 2002–31 December 2002 and 1 March 2005–28 February 2006, respectively).

**Table 1:** Maternal characteristics from birth records of preterm (< 37 weeks gestation) and full term singleton births in Georgia with an estimated date of conception during 1 January 2002 through 28 February 2006 [*n* (%)].

Maternal characteristic	Preterm birth (*n* = 47,321)	Full term birth (*n* = 464,337)	*p*-Value*
Residence in large metropolitan county	24,974 (43.2)	263,708 (47.2)	< 0.0001
Race
White	25,514 (65.4)	303,638 (53.9)	< 0.0001
African American	20,283 (30.4)	141,300 (42.9)
Asian	1,242 (3.5)	16,359 (2.6)
American Indian or Alaskan	86 (0.2)	972 (0.2)
Native Hawaii or Pacific	35 (0.1)	329 (0.1)
Multiracial	155 (0.4)	1,731 (0.3)
Education
Less than 9th grade	2,957 (6.3)	31,181 (6.7)	< 0.0001
9th–12th grade	9,441 (20.0)	76,342 (16.4)
High school graduate	15,452 (32.7)	138,322 (29.8)
Some college or higher	17,893 (37.8)	304,831 (44.1)
Missing	1,574 (3.3)	13,651 (2.9)
Tobacco use during pregnancy	4,578 (9.9)	33,632 (7.3)	< 0.0001
Year 2000 census tract-level poverty
< 6.4% of residents below poverty	13,247 (28.0)	155,622 (33.5)	< 0.0001
6.4%–14.1% of residents below poverty	14,838 (31.3)	153,917 (33.2)
> 14.1% of residents below poverty	19,236 (40.7)	154,800 (33.3)
*p-Value from chi-square test for the differences among all categories of each characteristic.			

**Figure 1:**
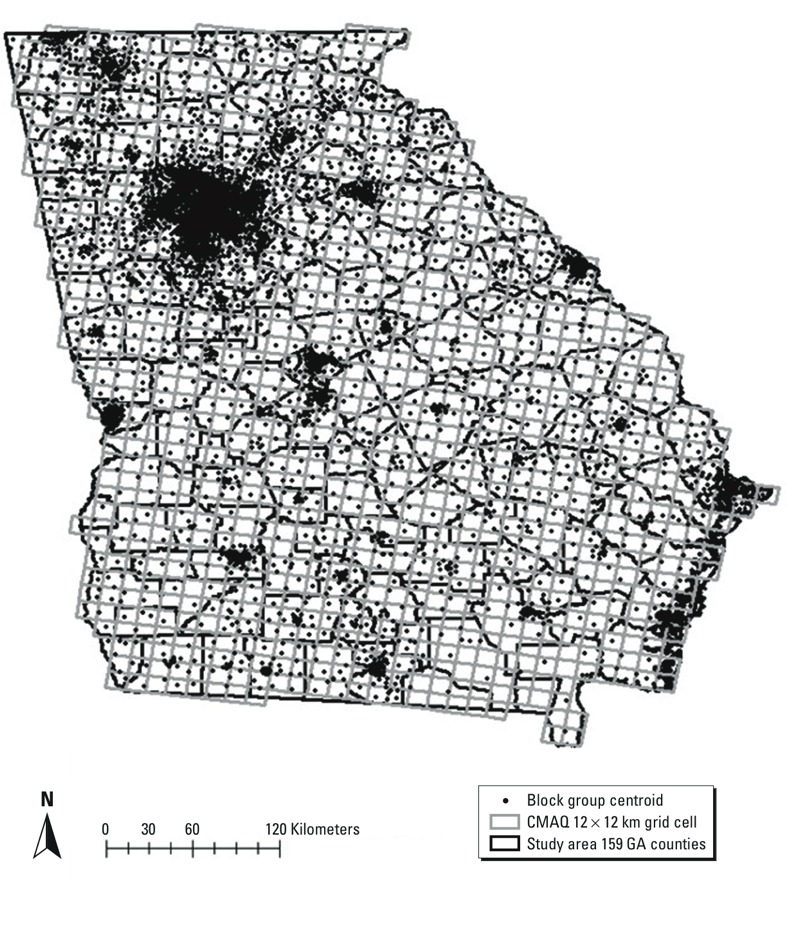
Map of the study area, which includes 159 counties in Georgia (thick black lines). CMAQ fusion 12 × 12 km grid cells (thin gray lines) are overlaid on the study area. Maternal census block–group centroids for residence at the time of delivery are indicated by black dots.

Maternal residence census blocks were linked with 942 CMAQ grid cells, and the average distance between maternal census block centroid and the CMAQ grid cell centroid was 4.6 km (Figure 1). Cross-validation results show the degree to which fusion of observations with CMAQ predictions improves prediction and reduces error compared with raw CMAQ outputs (Table 2). Eight-hour maximum O_3_ was predicted best (cross-validation *R* = 0.94, mean fractional error = 0.09) whereas 1-hr maximum SO_2_ was predicted most poorly due to limitations in modeling of coal combustion plumes (cross-validation *R* = 0.37, mean fractional error = 0.72). Compared to the unfused CMAQ estimates, the predictions from the CMAQ data fusion model resulted in improved cross-validation Pearson correlation coefficients and decreased mean fractional errors for all 11 pollutants considered. When data were not withheld, the Pearson’s correlations between daily measurements at monitoring stations and fused CMAQ estimates at grids where monitors are located were all ≥ 0.90 except for PM_10_ (0.87) and SO_2_ (0.72) (see Figure S3).

**Table 2:** Pearson correlation coefficients (*R*) and mean fractional error (MFE) of data fusion (DF) cross-validation predictions and CMAQ predictions of daily data at Georgia monitors, 2002–2008.

Pollutant	Number of monitors and frequency of measurements^*a*^	*R* DF	*R* CMAQ	MFE DF	MFE CMAQ
1-hr max CO (ppm)	5 daily	0.75	0.60	0.36	0.40
1-hr max NO_2_ (ppb)	7 daily	0.87	0.76	0.32	0.44
1-hr max SO_2_ (ppb)	14 daily	0.37	0.28	0.72	0.77
8-hr max O_3_ (ppb)	2 daily; 25 March–October^*b*^	0.94	0.80	0.09	0.18
24-hr ave PM_10_ (μg/m^3^)	2 daily; 1 1-in-3^*c*^; 17 1-in-6^*d*^	0.76	0.47	0.22	0.42
24-hr ave PM_2.5_ (μg/m^3^)	15 daily; 20 1-in-3; 7 1-in-6	0.89	0.56	0.16	0.38
24-hr ave SO_4_^2–^ (μg/m^3^)	2 daily; 3 1-in-3; 9 1-in-6	0.88	0.76	0.21	0.35
24-hr ave NO_3_^–^ (μg/m^3^)	2 daily; 3 1-in-3; 9 1-in-6	0.75	0.55	0.46	0.88
24-hr ave NH_4_^+^ (μg/m^3^)	2 daily; 3 1-in-3; 9 1-in-6	0.84	0.54	0.24	0.44
24-hr ave EC (μg/m^3^)	2 daily; 3 1-in-3; 9 1-in-6	0.73	0.66	0.37	0.48
24-hr ave OC (μg/m^3^)	2 daily; 3 1-in-3; 9 1-in-6	0.73	0.57	0.30	0.61
Abbreviations: ave, average; max, maximum. Leave-one-monitor-out cross-validation was used to calculate Pearson correlation coefficients between the predictions and the measurements from the monitor held out of the model fitting. Fractional error—the ratio of the error of the prediction to the measured concentration—was calculated for each monitor in the cross-validation, and these fractional errors were averaged to calculate the mean fractional error. ^***a***^Number of monitors in Georgia, including monitors that operated only for a subset of years, and frequency of measurements. ^***b***^Daily measurements during March through October. ^***c***^1-in-3: one 24-hr measurement every 3 days. ^***d***^1-in-6: one 24-hr measurement every 6 days.					

The distributions of daily pollutant concentrations are presented in Table 3. The IQRs of the total pregnancy average pollutant concentrations were for CO 0.06 ppm; NO_2_ 1.81 ppb; SO_2_ 1.59 ppb; O_3_ 6.43 ppb; PM_10_ 3.96 μg/m^3^; PM_2.5_ 2.01 μg/m^3^; SO_4_^2–^ 1.27 μg/m^3^; NO_3_^–^ 0.25 μg/m^3^; NH_4_^+^ 0.24 μg/m^3^; EC 0.14 μg/m^3^; and OC 0.36 μg/m^3^. (For distributions of trimester-specific and total pregnancy average pollutant concentrations, see Figure S4.) Correlations between average pollutant levels during pregnancy varied in magnitude, with correlation coefficients ranging from 0.03 (PM_10_ and NO_2_) to 0.95 (CO and NO_2_) (see Table S1).

**Table 3:** Selected percentiles of daily ambient air pollutant concentrations in Georgia, 2002–2006.

Pollutant (unit)	5th percentile	25th percentile	50th percentile	75th percentile	100th percentile
1-hr max CO (ppm)	0.12	0.18	0.24	0.33	0.96
1-hr max NO_2_ (ppb)	1.16	2.13	3.51	6.64	32.72
1-hr max SO_2_ (ppb)	0.58	1.71	3.58	6.70	25.41
8-hr max O_3_ (ppb)	23.58	32.67	40.88	51.14	75.06
24-hr ave PM_10_ (μg/m^3^)	8.64	14.79	20.82	28.03	54.38
24-hr ave PM_2.5_ (μg/m^3^)	4.91	8.07	11.44	15.86	33.67
24-hr ave SO_4_^2–^ (μg/m^3^)	1.23	2.06	3.13	4.85	30.10
24-hr ave NO_3_^–^ (μg/m^3^)	0.07	0.15	0.29	0.62	2.26
24-hr ave NH_4_^+^ (μg/m^3^)	0.36	0.63	0.94	1.39	3.49
24-hr ave EC (μg/m^3^)	0.15	0.28	0.42	0.67	2.08
24-hr ave OC (μg/m^3^)	0.80	1.37	2.03	2.99	7.95
Abbreviations: ave, average; max, maximum.					

**Figure 2:**
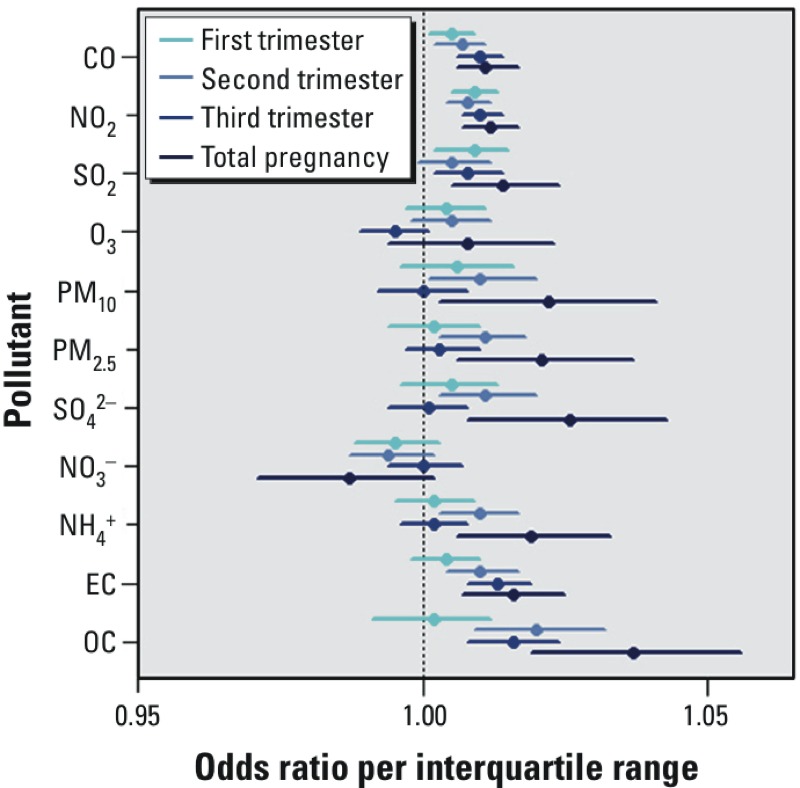
Statewide trimester-specific and total pregnancy–adjusted ORs and 95% confidence intervals per IQR increase in the concentrations of 11 pollutants. Models were adjusted for maternal education, race, smoking, and long-term trend using a natural cubic spline on conception date with 5 df (1 per year). IQRs: CO 0.06 ppm; NO_2_ 1.81 ppb; SO_2_ 1.59 ppb; O_3_ 6.43 ppb; PM_10_ 3.96 μg/m^3^; PM_2.5_ 2.01 μg/m^3^; SO_4_^2–^ 1.27 μg/m^3^; NO_3_^–^ 0.25 μg/m^3^; NH_4_^+^ 0.24 μg/m^3^; EC 0.14 μg/m^3^; OC 0.36 μg/m^3^.

Statewide ORs and 95% confidence intervals (CIs) for the risk of preterm birth per IQR increase in each of the 11 pollutants during the first, second, and third trimester and the full pregnancy are presented in Figure 2 (for numerical results, see Table S2). Statistically significant associations (*p* < 0.05) of preterm birth with IQR increase in first-trimester CO (OR = 1.005; 95% CI: 1.001, 1.009), NO_2_ (OR = 1.009; 95% CI: 1.005, 1.013), and SO_2_ (OR = 1.009; 95% CI: 1.002, 1.015) were observed. All but three pollutants (SO_2_, O_3_, and NO_3_^–^) were significantly associated with preterm birth during the second trimester, and in the third trimester CO, NO_2_, SO_2_, EC, and OC were significantly associated with increased risk of preterm birth. Associations for the total pregnancy period were frequently in the same direction as the trimester-specific estimates (Figure 2).

**Figure 3:**
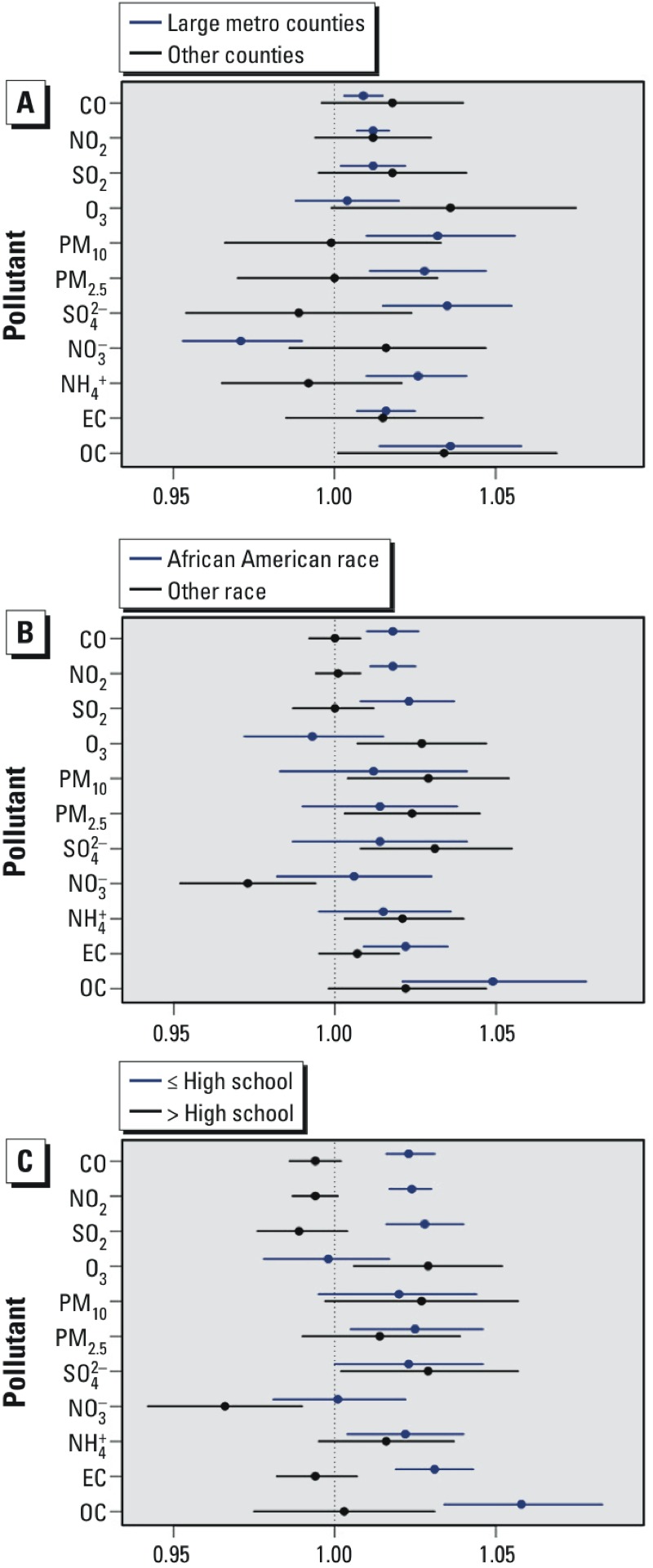
Total pregnancy–adjusted ORs and 95% confidence intervals per IQR increase in the concentrations of 11 pollutants, stratified according to (*A*) maternal residence in an urban county or maternal residence in a non-urban county; (*B*) African-American maternal race or non–African-American maternal race; (*C*) maternal education level high school or lower or maternal education level greater than high school. Models were adjusted for maternal education, race, smoking, and long-term trend using a natural cubic spline on conception date with 5 df (1 per year). IQRs: CO 0.06 ppm; NO_2_ 1.81 ppb; SO_2_ 1.59 ppb; O_3_ 6.43 ppb; PM_10_ 3.96 μg/m^3^; PM_2.5_ 2.01 μg/m^3^; SO_4_^2–^ 1.27 μg/m^3^; NO_3_^–^ 0.25 μg/m^3^; NH_4_^+^ 0.24 μg/m^3^; EC 0.14 μg/m^3^; OC 0.36 μg/m^3^.

Figure 3 shows total pregnancy results stratified by neighborhood urbanicity, maternal race, and maternal education level (for numerical results, as well as trimester-specific results, see Table S3). Trimester-specific associations between preterm birth and the pollutants that are markers of traffic emissions (CO, NO_2_, and EC) as well as SO_2_ (predominantly coal-burning power plant emissions) were frequently higher for mothers living in urban counties, for African-American mothers, and for mothers with lower education levels; however, the associations across the total pregnancy period were sometimes higher for mothers living in less urban counties. There was little suggestion of effect modification by percent of the census tract below poverty (results not shown).

Sensitivity analyses were performed by adding additional control to the regression spline on conception date (see Table S4). Associations with primary pollutants CO, NO_2_, and EC changed little across the sensitivity analyses, whereas the point estimates for SO_2_ increased with additional control. For secondary pollutants that have a more pronounced seasonal pattern, such as O_3_ and SO_4_, and for PM_10_ and PM_2.5_, which are a mix of primary and secondary particles, the confidence interval widths increased with additional control for time. Because we controlled for county via stratification, our analysis was largely informed by contrasts in pollutant concentrations over time, so a regression spline that controlled for season likely decreased the exposure variability that informed the estimation of the pollutant associations for these pollutants that have seasonal patterns.

## Discussion

In this study we used air quality characterizations obtained from fusing CMAQ outputs with monitoring data to investigate associations between 11 ambient air pollutants and the risk of preterm birth in Georgia. Except for EC during the first trimester, we observed statistically significant associations between the three primary markers of traffic pollution—CO, NO_2_, and EC—and increased risk of preterm birth across all exposure windows. Several previous studies have reported higher risks of preterm birth associated with higher concentrations of CO during pregnancy (Bell et al. 2007; Lin et al. 2004; Ritz et al. 2007), and a recent systematic review reported a pooled OR of 1.04 (95% CI: 1.02, 1.06) per 1-ppm increase in third-trimester CO (Stieb et al. 2012). However, the authors of recent systematic reviews also remarked on the substantial heterogeneity that exists among studies, which makes drawing conclusions difficult (Shah et al. 2011; Stieb et al. 2012).

In addition to the associations with traffic-related pollutants, we observed statistically significant positive associations with SO_2_ across all exposure windows except the second trimester. In their systematic review Stieb et al. (2012) note that results were not consistent across studies for SO_2_, a finding that might arise from the inability of a sparse monitoring system to capture the heterogeneous spatial distribution of SO_2_ concentrations due to the predominance of point emission sources from coal combustion stacks. Here we fused measurements from stationary monitors with CMAQ estimates to provide spatial information on SO_2_ concentrations, although the fusion approach provided only limited improvement in predictions of daily ground-level SO_2_ fields over unfused CMAQ predictions (cross-validation Pearson correlation coefficient improved from 0.28 to 0.37).

We also observed significant associations with OC during the second and third trimesters and total pregnancy, but the point estimate for the first-trimester association was close to null. There are fewer published results on the associations between PM_2.5_ components and preterm birth, although associations with OC and EC (averaged throughout the entire pregnancy) were reported in a Southern California study of preterm birth (Wilhelm et al. 2011). More work is needed to investigate associations between adverse birth outcomes and PM_2.5_ components.

We observed less evidence for associations with PM_10_, PM_2.5_, SO_4_^2–^, and NH_4_^+^. We observed significant second-trimester and total pregnancy associations (but not first- or third-trimester associations) with PM_10_, PM_2.5_, SO_4_^2–^, and NH_4_^+^; however, given that the trimester-average pairwise correlations among these pollutants ranged between 0.64 and 0.93 (see Table S1), it is not surprising that the ORs for these pollutants were similar. These second-trimester findings contrast with results recently published by Pereira et al. (2014), who reported point estimates that are mostly below the null for the second trimester exposures. Whether these inconsistencies are attributable to differences in study populations or research methods or to random error is difficult to surmise. In our study we did not observe associations with O_3_, which seems consistent with many previous findings; Shah et al. (2011) reported that none of the 10 papers published before 2010 found a significant association between O_3_ and risk of preterm birth (Shah et al. 2011).

Results from two California studies and one Canadian study suggest that neighborhood socioeconomic status (SES) and maternal education might modify relations between air pollution and birth outcomes in urban areas (Généreux et al. 2008; Ponce et al. 2005; Wilhelm and Ritz 2003). In our study we also investigated effect measure modification, and we observed larger ORs for the associations between outdoor air pollutant concentrations and preterm birth for women who lived in urban counties, women of African-American race, and women with lower education levels, although significance tests for these differences were not performed. These maternal characteristics were correlated, and given sample size limitations we did not attempt to disentangle the effects of one effect modifier from the others. Explanations underlying this effect modification may be complex and multifaceted; maternal stress, socioeconomic disparities and environmental justice issues affect health in many ways: access to health care, social patterning of health behaviors, and overall health status. Together, these factors could affect maternal responses to ambient air pollutant concentrations. Noncausal explanations, such as random error and differential exposure measurement error across maternal characteristics could also explain the observed associations.

Our study has several strengths. Foremost is the use of an innovative air quality model developed by combining CMAQ outputs with measurements from stationary monitors. This model provides complete spatial and temporal coverage over the study domain while reducing bias present in the CMAQ outputs (Mebust et al. 2003). Cross-validation analyses indicated that prediction errors decreased and Pearson correlation coefficients increased for all 11 pollutants considered when comparing the fused CMAQ estimates with unfused CMAQ estimates (Table 2).

Second, the analysis is based on a population of > 500,000 births, and the time-to-event statistical approach used to estimate third-trimester associations avoids the potential bias resulting from differences in the length of the third-trimester averaging period between preterm and full-term births (Lewis et al. 2011; O’Neill et al. 2003). For example, for a cohort of children conceived at the beginning of winter, those born preterm will have less exposure to pollutant concentrations during summer, when concentrations of several pollutants peak. The direction and magnitude of the potential bias therefore depend on the seasonality in both air pollution and the number of ongoing pregnancies in the population (Chang et al. 2013). Other approaches to overcome this bias include time-series studies that aggregate all ongoing at-risk pregnancies on each day (Darrow et al. 2009) and matched case–control studies that compare the same exposure period for cases and controls who are matched on conception date (Huynh et al. 2006).

This study has several limitations. Measurement errors arise when assigning air pollution levels to individual pregnancies. Pollutant levels were assumed to be spatially homogeneous within each 12-km grid; thus, fine-scale pollutant gradients, such as those associated with roadways, are not captured by this model. The magnitude of these spatial errors will vary by pollutant, with larger errors for spatially heterogeneous pollutants (such as CO, NO_2_, EC, and SO_2_) and smaller errors for secondary pollutants that tend to be more homogeneously distributed across space (such as O_3_ or SO_4_^2–^). Even so, we tended to observe the most consistent evidence for associations in our study with the traffic pollutants (CO, NO_2_, and EC), suggesting that if these measurement errors did cause bias to the null they were not so severe as to completely obfuscate our results. Maternal residential mobility is also a concern, because residence at the time of delivery was used to assign pollutant levels throughout pregnancy. A previous study conducted in Atlanta during 1993–1997 found that 22% of women moved residence during pregnancy, with approximately half moving within the same county (Miller et al. 2010). If this residential mobility was nondifferential with respect to both ambient air pollution levels and preterm birth, then the expected direction of bias would be toward the null, although we are uncertain if this was the case.

There are also concerns related to residual confounding, particularly surrounding socioeconomic position: Many socioeconomic factors are strong predictors of preterm birth, and these socioeconomic factors might be related to community-level air pollution concentrations (Blumenshine et al. 2010). We were unable to control for maternal medication status during pregnancy (Crowther et al. 2011), maternal illicit drug use (Parlier et al. 2014), and maternal stress during pregnancy (Dole et al. 2003). In our data we did not observe strong evidence of confounding by neighborhood-level socioeconomic variables (data not shown), although few individual-level socioeconomic covariates are routinely collected on birth records, which raises concerns about the adequacy of the confounding control. The authors of a previous study on air pollution and pregnancy outcomes concluded that confounding was a minor concern (Ritz et al. 2007), but more work is needed to explore this potential limitation, because the nature of confounding might differ from one population to another. Our inclusion of county-level fixed effects in the regression models limits comparisons to “within county,” although confounding might still exist among women in the same county. Finally, there are inaccuracies in the recording of gestational age on birth records (Bakketeig 1991; Parker and Schoendorf 2002), and consequently some preterm births were likely misclassified. If this misclassification was nondifferential with respect to air pollution levels, then bias is expected to be toward the null; however, as was the case for the errors due to residential mobility, we are uncertain about the nature of this measurement error.

In summary, we observed significant associations with several different air pollutants and preterm birth in our study. Consistent associations across different exposure windows were found for traffic pollutants and for SO_2_. For these pollutants, associations were of greater magnitude for women of African-American race and women with lower education levels. We also observed associations with OC during the second and third trimester and the total pregnancy, and we observed second-trimester and total pregnancy associations with particulate matter. Although the mechanisms underlying these associations are not yet clear, these effects merit further examination in other populations.

## Supplemental Material

(1.3 MB) PDF
