# Supplementary material for: Air Pollution and Preterm Birth in the U.S. State of Georgia (2002–2006): Associations with Concentrations of 11 Ambient Air Pollutants Estimated by Combining Community Multiscale Air Quality Model (CMAQ) Simulations with Stationary Monitor Measurements
Source: Environ Health Perspect. 2015 Oct 20;124(6):875–80. doi: 10.1289/ehp.1409651 (PMC4892915; doi:10.1289/ehp.1409651)
Supplement: (1.3 MB) PDF [file ehp.1409651.s001.acco.pdf]

Environ Health Perspect
DOI: 10.1289/ehp.1409651

Note to readers with disabilities: *EHP* strives to ensure that all journal content is accessible to all readers. However, some figures and Supplemental Material published in *EHP* articles may not conform to 508 standards due to the complexity of the information being presented. If you need assistance accessing journal content, please contact ehp508@niehs.nih.gov. Our staff will work with you to assess and meet your accessibility needs within 3 working days.

Supplemental Material

Air Pollution and Preterm Birth in the U.S. State of Georgia (2002–2006): Associations with Concentrations of 11 Ambient Air Pollutants Estimated by Combining Community Multiscale Air Quality Model (CMAQ) Simulations with Stationary Monitor Measurements

Hua Hao, Howard H. Chang, Heather A. Holmes, James A. Mulholland, Mitch Klein, Lyndsey A. Darrow, and Matthew J. Strickland

Table of Contents

Figure S1: Georgia locations of ambient air quality monitors with observations during 2002-2008. SEARCH monitors at Jefferson St. in Atlanta and at Yorkville west of Atlanta are labeled H, indicating a comprehensive set of pollutant measurements are available at these sites.

Figure S2: Data fusion flow diagram for $PM_{2.5}$ ($\mu g/m^3$) on 7/23/08. This method has been developed for fusing spatially sparse and temporally incomplete ambient monitor measurements and 12-km resolution chemical transport model (CMAQ) outputs to estimate daily ground-level air pollutant fields (manuscript under review). The data fusion approach uses spatial autocorrelation models to maximize the prediction of temporal variance and regression models to minimize annual mean and seasonal biases, providing daily estimates of spatial fields of air pollutant concentrations and uncertainties that are consistent with observations, emissions, and meteorology.

Figure S3: Scatterplots and Pearson correlation coefficients between land-based monitor daily measurements and daily fused CMAQ model estimates.
Figure S4: Boxplots of distributions of 11 pollutants by different exposure windows. T1: First trimester. T2: Second trimester. T3: Third trimester. Total: Total pregnancy. Boxes extend from the 25th to the 75th percentile, horizontal bars represent the median, whiskers extend 1.5 times the length of the interquartile range (IQR) above and below the 75th and 25th percentiles, respectively, and outliers are represented as points.
Table S1: Total pregnancy Pearson correlation coefficients among 11 ambient air pollutants, Georgia, U.S.A., for conceptions between 1 January 2002 and 28 February 2006.
Table S2: Adjusted ORs and 95% CIs for preterm birth per IQR increase in 11 ambient air pollutants in Georgia, U.S.A., for conceptions between 1 January 2002 and 28 February 2006.
Table S3: Stratum-specific adjusted ORs and 95% CIs for preterm birth per IQR increase in 11 ambient air pollutants in Georgia, U.S.A., for conceptions between 1 January 2002 and 28 February 2006.
Table S4: Sensitivity analysis of adjusted ORs for preterm birth per IQR increase in 11 ambient air pollutants throughout the entire pregnancy period for different amounts of smoothing on conception date (degrees of freedom=5, 9 and 17).[a]

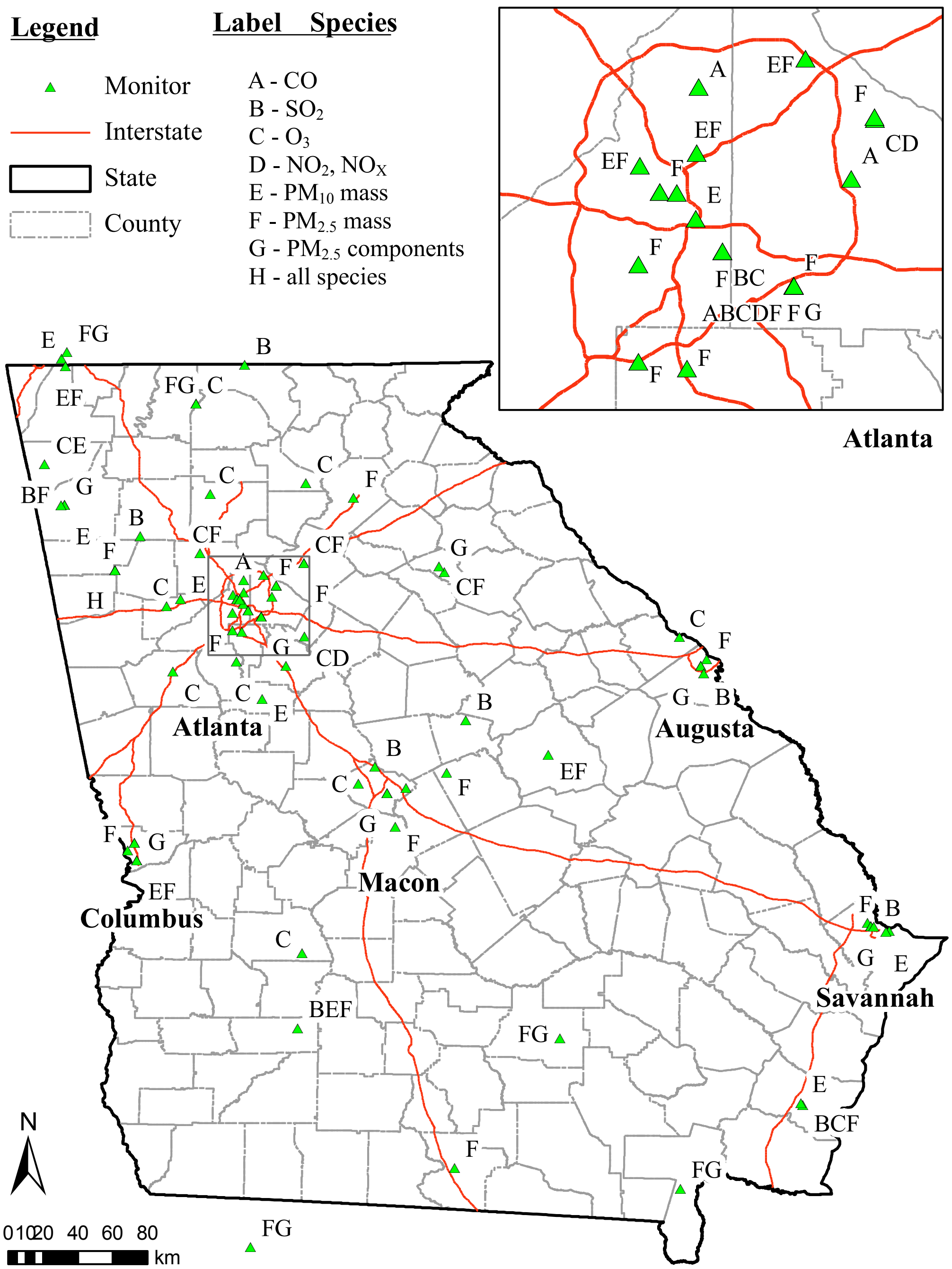

Figure S1: Georgia locations of ambient air quality monitors with observations during 2002-2008. SEARCH monitors at Jefferson St. in Atlanta and at Yorkville west of Atlanta are labeled H, indicating a comprehensive set of pollutant measurements are available at these sites.

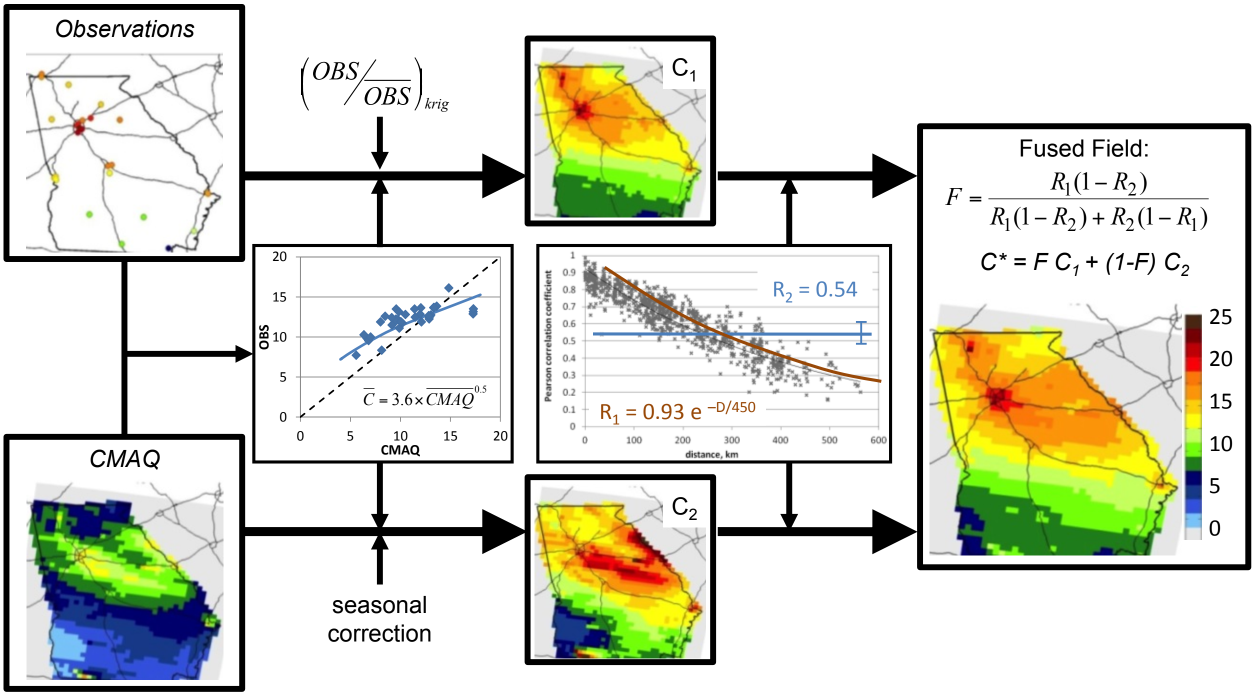

Figure S2: Data fusion flow diagram for $PM_{2.5}$ ($\mu g/m^3$) on 7/23/08. This method has been developed for fusing spatially sparse and temporally incomplete ambient monitor measurements and 12-km resolution chemical transport model (CMAQ) outputs to estimate daily ground-level air pollutant fields (manuscript under review). The data fusion approach uses spatial autocorrelation models to maximize the prediction of temporal variance and regression models to minimize annual mean and seasonal biases, providing daily estimates of spatial fields of air pollutant concentrations and uncertainties that are consistent with observations, emissions, and meteorology.

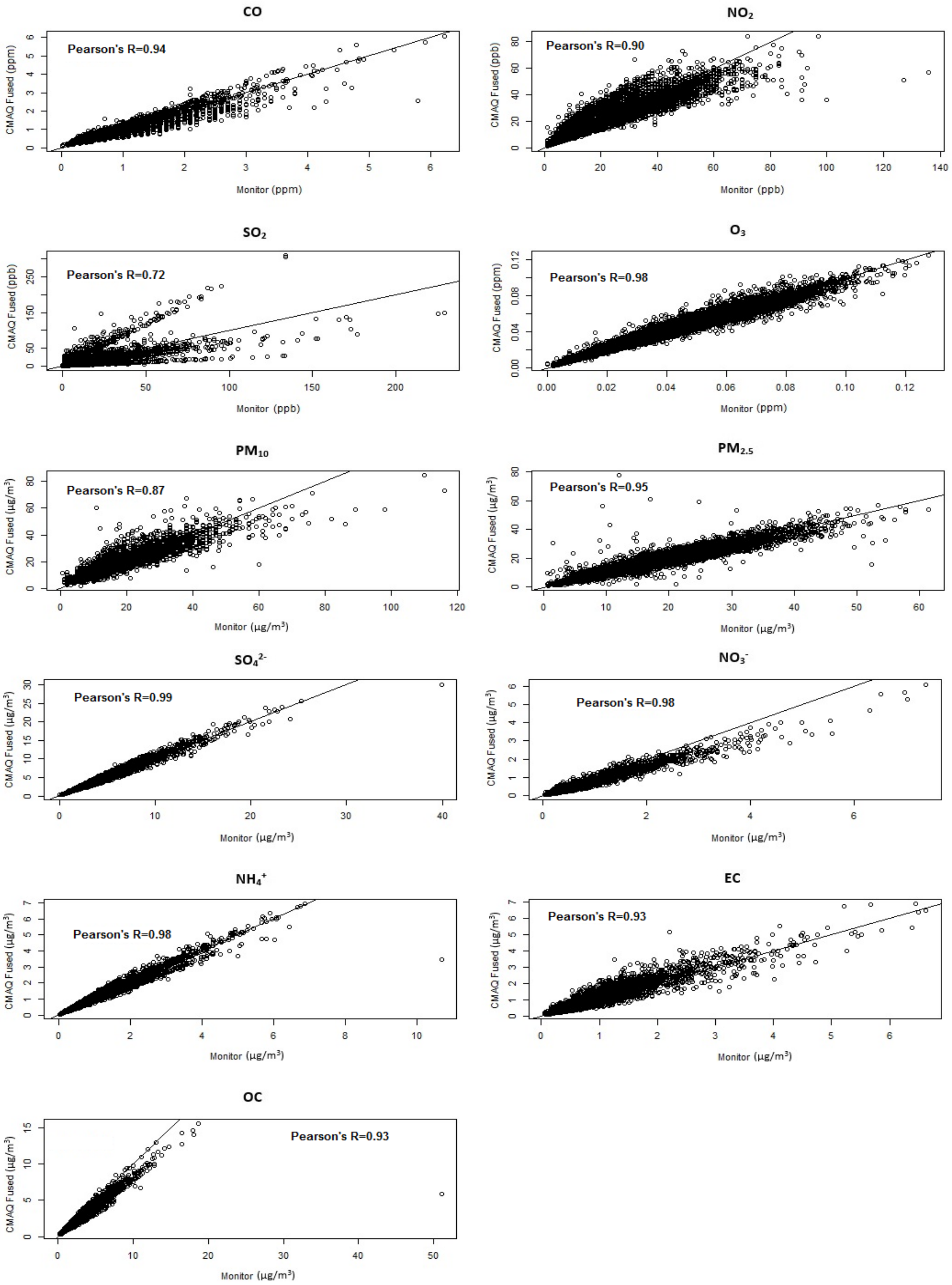

Figure S3: Scatterplots and Pearson correlation coefficients between land-based monitor daily measurements and daily fused CMAQ model estimates.

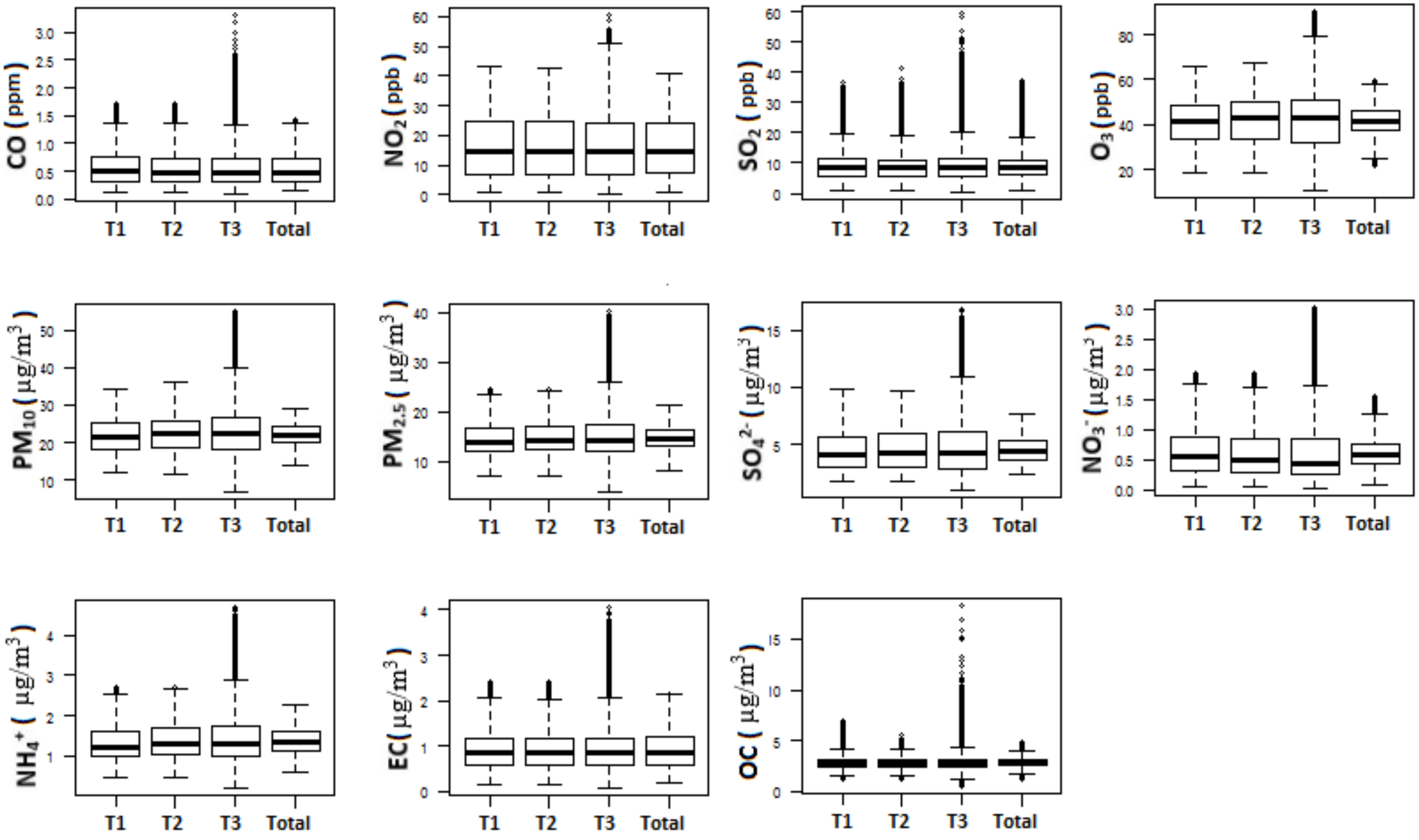

Figure S4: Boxplots of distributions of 11 pollutants by different exposure windows. T1: First trimester. T2: Second trimester. T3: Third trimester. Total: Total pregnancy. Boxes extend from the 25th to the 75th percentile, horizontal bars represent the median, whiskers extend 1.5 times the length of the interquartile range (IQR) above and below the 75th and 25th percentiles, respectively, and outliers are represented as points.

Table S1: Total pregnancy Pearson correlation coefficients among 11 ambient air pollutants, Georgia, U.S.A., for conceptions between 1 January 2002 and 28 February 2006.

Pollutant	CO	NO_2	SO_2	O_3	PM_{10}	$PM_{2.5}$	SO_4^{2-}	NO_3^-	NH_4^+	EC	OC
CO	1.00	0.95	0.75	-0.27	-0.04	0.47	0.21	0.52	0.42	0.91	0.72
NO_2		1.00	0.78	-0.19	0.03	0.51	0.23	0.45	0.44	0.93	0.71
SO_2			1.00	-0.18	-0.04	0.46	0.21	0.48	0.39	0.75	0.64
O_3				1.00	0.70	0.47	0.63	-0.64	0.44	-0.25	-0.06
PM_{10}					1.00	0.73	0.76	-0.61	0.65	0.07	0.18
$PM_{2.5}$						1.00	0.85	-0.12	0.94	0.55	0.59
SO_4^{2-}							1.00	-0.41	0.85	0.24	0.23
NO_3^-								1.00	-0.02	0.39	0.32
NH_4^+									1.00	0.44	0.43
EC										1.00	0.81
OC											1.00

Table S2: Adjusted ORs and 95% CIs for preterm birth per IQR increase in 11 ambient air pollutants in Georgia, U.S.A., for conceptions between 1 January 2002 and 28 February 2006.

Pollutant	Period of pregnancy	Adjusted OR (95% CI)
CO	First Trimester	1.005 (1.001, 1.009)
	Second Trimester	1.007 (1.002, 1.011)
	Third Trimester	1.010 (1.006, 1.014)
	Total Pregnancy	1.011 (1.006, 1.017)
NO_2	First Trimester	1.009 (1.005, 1.013)
	Second Trimester	1.008 (1.004, 1.012)
	Third Trimester	1.010 (1.007, 1.014)
	Total Pregnancy	1.012 (1.007, 1.017)
SO_2	First Trimester	1.009 (1.002, 1.015)
	Second Trimester	1.005 (0.999, 1.012)
	Third Trimester	1.008 (1.002, 1.014)
	Total Pregnancy	1.014 (1.005, 1.024)
O_3	First Trimester	1.004 (0.997, 1.011)
	Second Trimester	1.005 (0.998, 1.012)
	Third Trimester	0.995 (0.989, 1.001)
	Total Pregnancy	1.008 (0.994, 1.023)
PM_{10}	First Trimester	1.006 (0.996, 1.016)
	Second Trimester	1.010 (1.001, 1.020)
	Third Trimester	1.000 (0.992, 1.008)
	Total Pregnancy	1.022 (1.003, 1.041)
$PM_{2.5}$	First Trimester	1.002 (0.994, 1.010)
	Second Trimester	1.011 (1.003, 1.018)
	Third Trimester	1.003 (0.997, 1.010)
	Total Pregnancy	1.021 (1.006, 1.037)
SO_4^{2-}	First Trimester	1.005 (0.996, 1.013)
	Second Trimester	1.011 (1.003, 1.020)
	Third Trimester	1.001 (0.994, 1.008)
	Total Pregnancy	1.026 (1.008, 1.043)
NO_3^-	First Trimester	0.995 (0.988, 1.003)
	Second Trimester	0.994 (0.987, 1.002)
	Third Trimester	1.000 (0.994, 1.007)
	Total Pregnancy	0.987 (0.971, 1.002)
NH_4^+	First Trimester	1.002 (0.995, 1.009)
	Second Trimester	1.010 (1.003, 1.017)
	Third Trimester	1.002 (0.996, 1.008)
	Total Pregnancy	1.019 (1.006, 1.033)
EC	First Trimester	1.004 (0.998, 1.010)
	Second Trimester	1.010 (1.004, 1.017)
	Third Trimester	1.013 (1.008, 1.019)
	Total Pregnancy	1.016 (1.007, 1.025)
OC	First Trimester	1.002 (0.991, 1.012)
	Second Trimester	1.020 (1.009, 1.032)
	Third Trimester	1.016 (1.008, 1.024)
	Total Pregnancy	1.037 (1.019, 1.056)

Models adjusted for maternal education, race, smoking, and long-term trend using a natural cubic spline on conception date with 5 degrees of freedom (one per year). Interquartile ranges: CO 0.06 ppm; NO_2 1.81 ppb; SO_2 1.59 ppb; O_3 6.43 ppb; PM_{10} 3.96 µg/m^3; $PM_{2.5}$ 2.01 µg/m^3; SO_4^{2-} 1.27 µg/m^3; NO_3^- 0.25 µg/m^3; NH_4^+ 0.24 µg/m^3; EC 0.14 µg/m^3; OC 0.36 µg/m^3.

Table S3: Stratum-specific adjusted ORs and 95% CIs for preterm birth per IQR increase in 11 ambient air pollutants in Georgia, U.S.A., for conceptions between 1 January 2002 and 28 February 2006.[a]

	**Maternal education**		**Maternal Race**		**Maternal County**	
**Pollutant and time period**	**≤ High school**	**> High school**	**African American**	**Other race**	**Large metropolitan**	**Medium, small, and non-metropolitan**
**CO**						
1st Trimester	1.010 (1.004, 1.016)	0.996 (0.989, 1.002)	1.012 (1.005, 1.018)	0.996 (0.990, 1.002)	1.005 (1.000, 1.010)	1.000 (0.990, 1.009)
2nd Trimester	1.014 (1.008, 1.020)	0.995 (0.988, 1.001)	1.013 (1.007, 1.020)	0.998 (0.992, 1.004)	1.006 (1.001, 1.011)	1.003 (0.994, 1.012)
3rd Trimester	1.015 (1.010, 1.020)	1.002 (0.996, 1.008)	1.012 (1.006, 1.017)	1.007 (1.001, 1.012)	1.011 (1.007, 1.016)	1.000 (0.991, 1.009)
Total Pregnancy	1.023 (1.016, 1.031)	0.994 (0.986, 1.002)	1.018 (1.010, 1.026)	1.000 (0.992, 1.008)	1.009 (1.003, 1.015)	1.018 (0.996, 1.040)
**NO_2**						
1st Trimester	1.014 (1.009, 1.020)	0.998 (0.992, 1.004)	1.013 (1.006, 1.019)	1.002 (0.996, 1.008)	1.012 (1.007, 1.017)	0.999 (0.991, 1.007)
2nd Trimester	1.015 (1.010, 1.021)	0.994 (0.988, 1.000)	1.016 (1.010, 1.022)	0.996 (0.990, 1.001)	1.011 (1.006, 1.015)	0.998 (0.990, 1.006)
3rd Trimester	1.017 (1.012, 1.023)	0.998 (0.992, 1.003)	1.014 (1.009, 1.020)	1.003 (0.997, 1.008)	1.015 (1.011, 1.020)	0.996 (0.989, 1.003)
Total Pregnancy	1.024 (1.017, 1.030)	0.994 (0.987, 1.001)	1.018 (1.011, 1.025)	1.001 (0.994, 1.008)	1.012 (1.007, 1.017)	1.012 (0.994, 1.030)
**SO_2**						
1st Trimester	1.012 (1.004, 1.021)	1.000 (0.990, 1.010)	1.011 (1.001, 1.021)	1.002 (0.993, 1.011)	1.008 (1.000, 1.016)	1.007 (0.996, 1.018)
2nd Trimester	1.011 (1.002, 1.020)	0.993 (0.983, 1.004)	1.016 (1.006, 1.026)	0.992 (0.983, 1.001)	1.005 (0.996, 1.013)	1.004 (0.993, 1.015)
3rd Trimester	1.016 (1.008, 1.023)	0.994 (0.985, 1.003)	1.011 (1.002, 1.019)	1.002 (0.994, 1.010)	1.010 (1.003, 1.018)	1.001 (0.991, 1.010)
Total Pregnancy	1.028 (1.016, 1.040)	0.989 (0.976, 1.004)	1.023 (1.008, 1.037)	1.000 (0.987, 1.012)	1.012 (1.002, 1.022)	1.018 (0.995, 1.041)
**O_3**						
1st Trimester	1.001 (0.992, 1.010)	1.009 (0.998, 1.019)	0.995 (0.984, 1.005)	1.012 (1.002, 1.021)	1.007 (0.999, 1.015)	0.997 (0.983, 1.011)
2nd Trimester	1.002 (0.993, 1.011)	1.011 (1.000, 1.021)	0.998 (0.987, 1.008)	1.012 (1.003, 1.021)	1.004 (0.996, 1.012)	1.009 (0.995, 1.023)
3rd Trimester	0.997 (0.989, 1.005)	0.993 (0.984, 1.003)	0.998 (0.989, 1.008)	0.993 (0.985, 1.001)	0.989 (0.982, 0.996)	1.009 (0.997, 1.022)
Total Pregnancy	0.998 (0.978, 1.017)	1.029 (1.006, 1.052)	0.993 (0.972, 1.015)	1.027 (1.007, 1.047)	1.004 (0.988, 1.020)	1.036 (0.999, 1.075)
**PM_{10}**						
1st Trimester	1.008 (0.995, 1.021)	1.003 (0.987, 1.019)	0.998 (0.983, 1.014)	1.010 (0.997, 1.023)	1.012 (0.999, 1.026)	0.997 (0.982, 1.013)
2nd Trimester	1.009 (0.997, 1.021)	1.013 (0.999, 1.028)	1.001 (0.986, 1.015)	1.017 (1.005, 1.029)	1.019 (1.007, 1.030)	0.998 (0.984, 1.012)
3rd Trimester	1.001 (0.991, 1.012)	0.998 (0.986, 1.011)	1,004 (0.992, 1.017)	0.997 (0.986, 1.007)	0.993 (0.982, 1.003)	1.010 (0.998, 1.023)
Total Pregnancy	1.020 (0.995, 1.044)	1.027 (0.997, 1.057)	1.012 (0.983, 1.041)	1.029 (1.004, 1.054)	1.032 (1.010, 1.018)	0.999 (0.966, 1.033)
**$PM_{2.5}$**						
1st Trimester	1.005 (0.995, 1.015)	0.997 (0.985, 1.009)	0.998 (0.986, 1.011)	1.003 (0.993, 1.013)	1.008 (0.998, 1.018)	0.993 (0.980, 1.007)
2nd Trimester	1.010 (1.000, 1.020)	1.012 (1.000, 1.024)	1.002 (0.991, 1.014)	1.016 (1.006, 1.025)	1.017 (1.008, 1.026)	0.998 (0.985, 1.011)

3rd Trimester	1.006 (0.998, 1.014)	0.999 (0.989, 1.009)	1.008 (0.998, 1.018)	0.999 (0.991, 1.008)	0.998 (0.990, 1.006)	1.012 (1.001, 1.023)
Total Pregnancy	1.025 (1.005, 1.046)	1.014 (0.990, 1.039)	1.014 (0.990, 1.038)	1.024 (1.003, 1.045)	1.028 (1.011, 1.047)	1.000 (0.970, 1.032)
**SO_4^{2-}**						
1st Trimester	1.008 (0.997, 1.019)	1.000 (0.987, 1.013)	1.000 (0.987, 1.013)	1.007 (0.996, 1.018)	1.009 (0.999, 1.019)	0.995 (0.981, 1.010)
2nd Trimester	1.009 (0.998, 1.019)	1.015 (1.002, 1.028)	1.002 (0.989, 1.015)	1.017 (1.006, 1.028)	1.018 (1.008, 1.028)	0.995 (0.981, 1.010)
3rd Trimester	1.001 (0.992, 1.011)	1.000 (0.988, 1.011)	1.005 (0.994, 1.016)	0.997 (0.988, 1.006)	0.995 (0.987, 1.004)	1.009 (0.997, 1.022)
Total Pregnancy	1.023 (1.000, 1.046)	1.029 (1.002, 1.057)	1.014 (0.987, 1.041)	1.031 (1.008, 1.055)	1.035 (1.015, 1.055)	0.989 (0.954, 1.024)
**NO_3^-**						
1st Trimester	0.995 (0.985, 1.005)	0.995 (0.984, 1.007)	1.003 (0.991, 1.014)	0.991 (0.981, 1.001)	0.989 (0.980, 0.999)	1.003 (0.991, 1.015)
2nd Trimester	0.999 (0.989, 1.009)	0.988 (0.976, 1.000)	1.004 (0.992, 1.016)	0.988 (0.978, 0.998)	0.988 (0.979, 0.998)	1.002 (0.990, 1.014)
3rd Trimester	1.004 (0.995, 1.013)	0.995 (0.985, 1.006)	1.001 (0.990, 1.011)	1.000 (0.991, 1.009)	1.003 (0.994, 1.011)	0.995 (0.984, 1.006)
Total Pregnancy	1.001 (0.981, 1.022)	0.966 (0.942, 0.990)	1.006 (0.982, 1.030)	0.973 (0.952, 0.994)	0.971 (0.953, 0.990)	1.016 (0.986, 1.047)
**NH_4^+**						
1st Trimester	1.006 (0.997, 1.015)	0.996 (0.986, 1.007)	1.000 (0.989, 1.011)	1.003 (0.994, 1.012)	1.077 (0.998, 1.015)	0.993 (0.980, 1.005)
2nd Trimester	1.009 (1.000, 1.017)	1.012 (1.002, 1.022)	1.003 (0.993, 1.014)	1.014 (1.005, 1.023)	1.015 (1.007, 1.023)	0.998 (0.986, 1.009)
3rd Trimester	1.003 (0.996, 1.011)	1.000 (0.991, 1.009)	1.007 (0.998, 1.016)	0.998 (0.991, 1.005)	0.998 (0.991, 1.005)	1.010 (0.999, 1.020)
Total Pregnancy	1.022 (1.004, 1.040)	1.016 (0.995, 1.037)	1.015 (0.995, 1.036)	1.021 (1.003, 1.040)	1.026 (1.010, 1.041)	0.992 (0.965, 1.016)
**EC**						
1st Trimester	1.010 (1.002, 1.019)	0.993 (0.983, 1.002)	1.014 (1.005, 1.024)	0.993 (0.984, 1.001)	1.007 (0.999, 1.014)	1.001 (0.990, 1.013)
2nd Trimester	1.019 (1.010, 1.027)	0.996 (0.987, 1.006)	1.014 (1.005, 1.024)	1.005 (0.996, 1.014)	1.013 (1.006, 1.021)	1.005 (0.994, 1.016)
3rd Trimester	1.017 (1.010, 1.024)	1.006 (0.997, 1.014)	1.013 (1.005, 1.021)	1.012 (1.004, 1.020)	1.019 (1.012, 1.026)	1.000 (0.990, 1.010)
Total Pregnancy	1.031 (1.019, 1.043)	0.994 (0.982, 1.007)	1.022 (1.009, 1.035)	1.007 (0.995, 1.020)	1.016 (1.007, 1.025)	1.015 (0.985, 1.046)
**OC**						
1st Trimester	1.003 (0.990, 1.016)	0.999 (0.982, 1.015)	1.015 (1.000, 1.031)	0.989 (0.975, 1.003)	1.006 (0.990, 1.022)	1.002 (0.988, 1.016)
2nd Trimester	1.032 (1.018, 1.047)	1.000 (0.983, 1.018)	1.021 (1.004, 1.038)	1.018 (1.003, 1.033)	1.024 (1.008, 1.040)	1.013 (0.997, 1.028)
3rd Trimester	1.024 (1.013, 1.034)	1.003 (0.989, 1.016)	1.015 (1.003, 1.027)	1.015 (1.004, 1.027)	1.016 (1.004, 1.028)	1.013 (1.001, 1.024)
Total Pregnancy	1.058 (1.034, 1.083)	1.003 (0.975, 1.031)	1.049 (1.021, 1.078)	1.022 (0.998, 1.047)	1.036 (1.014, 1.058)	1.034 (1.001, 1.069)

[a]Results from the total pregnancy models are shown in Figure 3 in the main text.

Models adjusted for maternal education, race, smoking, and long-term trend using a natural cubic spline on conception date with 5 degrees of freedom (one per year). Interquartile ranges: CO 0.06 ppm; NO_2 1.81 ppb; SO_2 1.59 ppb; O_3 6.43 ppb; PM_{10} 3.96 µg/m^3; $PM_{2.5}$ 2.01 µg/m^3; SO_4^{2-} 1.27 µg/m^3; NO_3^- 0.25 µg/m^3; NH_4^+ 0.24 µg/m^3; EC 0.14 µg/m^3; OC 0.36 µg/m^3.

Table S4: Sensitivity analysis of adjusted ORs for preterm birth per IQR increase in 11 ambient air pollutants throughout the entire pregnancy period for different amounts of smoothing on conception date (degrees of freedom=5, 9 and 17).[a]

Pollutant	Degrees of freedom	Adjusted OR (95% CI)
CO	5	1.011 (1.006, 1.017)
CO	9	1.011 (1.006, 1.017)
CO	17	1.014 (1.008, 1.020)
NO_2	5	1.012 (1.007, 1.017)
NO_2	9	1.013 (1.008, 1.018)
NO_2	17	1.015 (1.010, 1.020)
SO_2	5	1.014 (1.005, 1.023)
SO_2	9	1.018 (1.009, 1.028)
SO_2	17	1.022 (1.012, 1.032)
O_3	5	1.008 (0.993, 1.023)
O_3	9	1.014 (0.996, 1.033)
O_3	17	0.996 (0.968, 1.025)
PM_{10}	5	1.022 (1.003, 1.041)
PM_{10}	9	1.052 (1.018, 1.088)
PM_{10}	17	1.093 (1.026, 1.165)
$PM_{2.5}$	5	1.021 (1.006, 1.037)
$PM_{2.5}$	9	1.044 (1.021, 1.069)
$PM_{2.5}$	17	1.051 (1.021, 1.081)
SO_4^{2-}	5	1.026 (1.008, 1.043)
SO_4^{2-}	9	1.059 (1.030, 1.088)
SO_4^{2-}	17	1.104 (1.056, 1.154)
NO_3^-	5	0.986 (0.971, 1.002)
NO_3^-	9	0.980 (0.958, 1.002)
NO_3^-	17	1.008 (0.967, 1.051)
NH_4^+	5	1.019 (1.006, 1.033)
NH_4^+	9	1.040 (1.020, 1.061)
NH_4^+	17	1.047 (1.021, 1.073)
EC	5	1.016 (1.007, 1.025)
EC	9	1.015 (1.006, 1.025)
EC	17	1.020 (1.010, 1.030)
OC	5	1.037 (1.019, 1.056)
OC	9	1.037 (1.017, 1.057)
OC	17	1.047 (1.026, 1.069)

[a]Estimates from the model with 5 degrees of freedom are shown in Figure 2 in the main text.
Models adjusted for maternal education, race, and smoking. Interquartile ranges: CO 0.06 ppm; NO_2 1.81 ppb; SO_2 1.59 ppb; O_3 6.43 ppb; PM_{10} 3.96 μg/m^3; $PM_{2.5}$ 2.01 μg/m^3; SO_4^{2-} 1.27 μg/m^3; NO_3^- 0.25 μg/m^3; NH_4^+ 0.24 μg/m^3; EC 0.14 μg/m^3; OC 0.36 μg/m^3.
